# Removal of Cr(VI) from Aqueous Solution by Polypyrrole/Hollow Mesoporous Silica Particles

**DOI:** 10.3390/nano10040686

**Published:** 2020-04-05

**Authors:** Linlin Du, Peng Gao, Yuanli Liu, Tsuyoshi Minami, Chuanbai Yu

**Affiliations:** 1 Guangxi Key Laboratory of Optical and Electronic Materials and Devices, College of Materials Science and Engineering, Guilin University of Technology, Guilin 541004, China; linlindu0916@163.com (L.D.); pgaoglut@163.com (P.G.); 2 Institute of Industrial Science, the University of Tokyo, 4-6-1 Komaba, Meguro-ku 153-8505, Tokyo, Japan; tminami@iis.u-tokyo.ac.jp

**Keywords:** polypyrrole/hollow mesoporous silica particle, chromium(Cr), adsorption

## Abstract

The removal of Cr(VI) in wastewater plays an important role in human health and environment. In this work, polypyrrole/hollow mesoporous silica particle (PPy/HMSNs) adsorbents have been newly synthesized by in-situ polymerization, which prevent the aggregation of pyrrole in the process of polymerization and exhibit highly selective and powerful adsorption ability for Cr(VI). The adsorption process was in good agreement with the quasi-second-order kinetic model and the Langmuir isotherm model. And the maximum adsorption capacity of Cr(VI) was 322 mg/g at 25 °C. Moreover, the removal rate of Cr(VI) by PPy/HMSNs was ~100% in a number of binary systems, such as Cl^−^/Cr(VI), NO_3_^−^/Cr(VI), SO_4_^2−^/Cr(VI), Zn^2+^/Cr(VI), Fe^3+^/Cr(VI), Sn^4+^/Cr(VI), and Cu^2+^/Cr(VI). Thus, the PPy/HMSNs adsorbents have great potential for the removal of Cr(VI) in wastewater.

## 1. Introduction

Water pollution has become extremely serious with continuous industrial development. Heavy metals have become one of the most damaging water pollutants owing to their persistent toxicities and difficult degradation properties. For example, a large amount of chromium wastewater is discharged into the environment because Cr is used in many industrial processes, such as metallurgy and electroplating, and in paint [1,2]. Cr generally exists in both Cr(III) and Cr(VI) states in aqueous media. Cr(VI) is highly toxic and easily deposited in the human body through the food chain. In addition, Cr(VI) is considered to be a hazardous substance [3,4,5]. Therefore, efficient removal of Cr(VI) from water has become one of the hot issues in the field of environmental and analytical chemistry, and industrial process engineering.

The adsorption method possesses the advantages of having no secondary pollution, low cost, and simple preparation as a removal method for Cr(VI), and has gained much attention [6,7,8]. However, the type of adsorbent is very important when considering adsorption effects. At present, most of the adsorbents are organic–inorganic composites [9,10,11]. They not only have the adsorption sites provided by organic materials but also have the skeleton support of inorganic materials. An organic ligand, polypyrrole (PPy), has been widely used for adsorption owing to its good biocompatibility, ion exchange, and good conductivity. However, improvement of its adsorption effect by regulating PPy agglomeration during the polymerization process is a still challenging project [12,13]. Therefore, the adsorption effect of PPy for Cr(VI) can be substantially improved by solving the agglomeration issues. To this end, our attention has been focused on hollow mesoporous materials composed of hollow cavities and mesoporous shells of various sizes, because those larger specific surface areas, lower densities, and higher permeability offer great potential as adsorption materials [14,15].

This study reports on an inorganic–organic hybrid absorbent, polypyrrole/hollow mesoporous silica particles (PPy/HMSNs). To maximize its adsorption properties for Cr(VI), we optimized parameters such as pH, adsorbent dose, and the pyrrole ratio in PPy/HMSNs. Moreover, adsorption kinetics, isotherms, thermodynamics, and mechanisms have been fully investigated.

## 2. Materials and Methods

Materials: styrene (St), methyl methacrylate (MMA), acrylic acid (AA), sodium dodecylbenzene sulfonate (SDBS), tetraethyl silicate (TEOS), pyrrole, ferric chloride hexahydrate (FeCl_3_·6H_2_O), potassium dichromate (K_2_Cr_2_O_7_), alkaline aluminum oxide, sodium chloride (NaCl), sodium nitrate (NaNO_3_), sodium sulfate (Na_2_SO_4_), zinc chloride (ZnCl_2_), stannic chloride (SnCl_4_·5H_2_O), copper nitrate trihydrate (Cu(NO_3_)_2_·3H_2_O), sulfuric acid (H_2_SO_4_), phosphoric acid (H_3_PO_4_), diphenyl carbamide (C_13_H_14_N_4_O), acetone (C_3_H_6_O), hydrochloric acid (HCl), and sodium hydroxide (NaOH) were purchased from Aladdin (China, Shanghai). Ammonium bicarbonate (NH_4_HCO_3_), ammonium persulfate (APS), and polyethylene oxide-polypropylene oxide-polyethylene oxide (P123) were purchased form Sigma-Aldrich (Missouri, MO, USA). All chemicals were of analytical grade. St was purified by alkaline aluminum oxide column chromatography, and all other chemicals were used without further purification.

**Figure 1:**
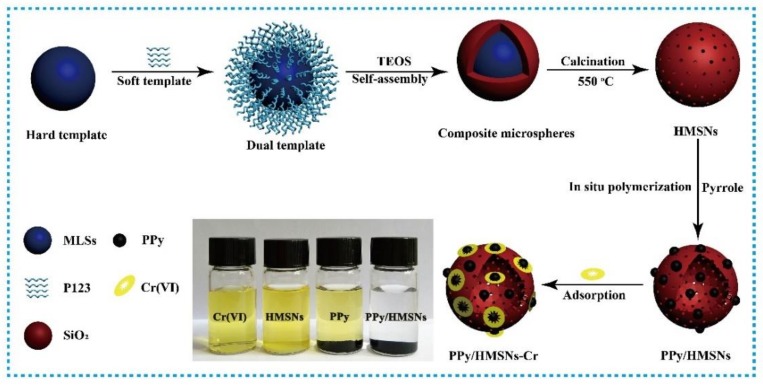
The preparation flow chart of polypyrrole/hollow mesoporous silica particles (PPy/HMSNs) and the adsorption effect for Cr(VI), and photographs of an aqueous solution of Cr(VI) before and after mixing with hollow mesoporous silica particles (HMSNs), polypyrrole (PPy), and PPy/HMSNs.

Synthesis of PPy/HMSNs: the preparation scheme of the hybrid is illustrated in Figure 1. First, P(St-MMA-AA) monodisperse core-shell spheres (MLSs) were prepared by emulsion polymerization. Next, 30.0 g MLSs was added to the mixed solution of 35.0 g water, 0.5 g P123, and 10.0 mL concentrated hydrochloric acid and stirred for 4 h at 30 °C. Next, 4.25 g TEOS and the above solution were mixed and stirred for 24 h. Then, the mixture was transferred to the high-pressure reactor and crystallized at 100 °C for 24 h. After washing with water, the solid was dried at 50 °C for 10 h. Hollow mesoporous silica particles (HMSNs) were obtained through calcining of the above sample at 550 °C for 300 min. Finally, HMSNs (1.0 g) and pyrrole monomer were dispersed in water with 30 min of sonication, and the content of pyrrole was 10%, 20%, 30%, 40%, 50%, 60%, 70%, 80%, 90%, and 100% of HMSNs. One hundred milliliters of FeCl_3_·6H_2_O solution (0.5 M HCl) was added to the above solution and stirred for 12 h. The mixed solution was washed with anhydrous ethanol and water 3 times, and dried under vacuum at 60 °C for 12 h to prepare PPy/HMSNs composite material. The PPy/HMSNs composite with pyrrole content of 60 wt % was used for the experiments. For more adsorption experiment details, see the Supplementary Materials.

Characterization: The samples were investigated by Fourier transform infrared (FTIR, Thermo Nexus 470, Massachusetts, MA, USA), thermal gravimetric analyzer (TGA, TA Q500, Delaware, DE, USA), X-ray diffraction (X’Pert PRO, Almelo, Netherlands), scanning electron microscopy (SEM, S-4800, Tokyo, Japan), transmission electron microscope (TEM, JEOLJEM-2100F, Tokyo, Japan), energy dispersive spectrometer (EDS, S-4800, Tokyo, Japan), and N_2_ adsorption-desorption (Quantachrome, Autosorb-SI, Florida, FL, USA). The Cr (VI) concentration in solution was tested using a PerkinElmer instrument (Platinum Elmer Enterprise Management Co., Ltd. Massachusetts, MA, USA), and the determination method of Cr (VI) solutions concentration is shown in Figure S1 and Supplementary Materials.

## 3. Results and Discussion

**Figure 2:**
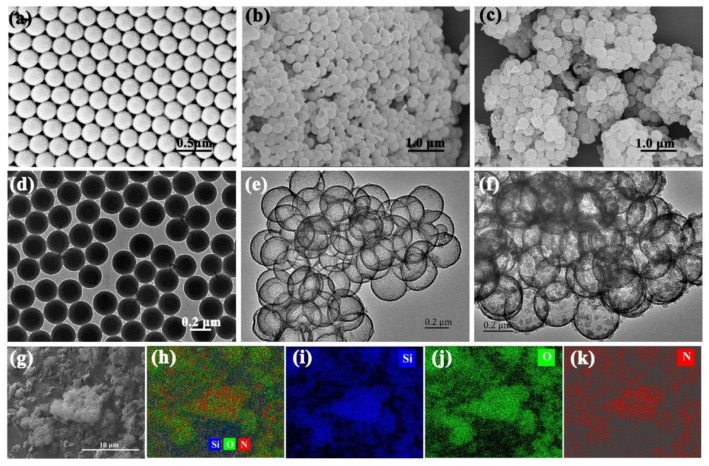
SEM and TEM images of monodisperse core-shell spheres (MLSs) (**a**,**d**), HMSNs (**b**,**e**) and PPy/HMSNs (**c**,**f**,**g**), and EDS mapping images of overlapped Si, O, N (**h**), Si (**i**), O (**j**), and N (**k**) of PPy/HMSNs.

To characterize the hybrid material, we firstly measured SEM and TEM images of MLSs, HMSNs, and PPy/HMSNs (Figure 2a–f). As expected, the MLSs possessed a core-shell structure with good sphericity, monodispersity, and uniformity. The particle size was estimated to be 262 ± 8 nm. On the other hand, the image of HMSNs showed broken ball-like structures, meaning that HMSNs have hollow and spherical structures. The average particle size was 298 ± 7 nm with a shell thickness of 18 nm, which was larger than that of MLSs. Moreover, the roughened surface of PPy/HMSNs had many particles. Thus, we can conclude that silica was successfully wrapped on the surface of MLSs. The energy-dispersive X-ray spectroscopy (EDS) mapping images (Figure 2g–k) of PPy/HMSNs displayed that the N element of pyrrole distributed uniformly on HMSNs. The results indicate that PPy was successfully and uniformly wrapped on the surface of HMSNs. The pyrrole NH is protonated (i.e., positively charged) during polymerization, while the surface of HMSNs contained a large number of deprotonated hydroxyl groups (i.e., negatively charged); thus the pyrrole monomers should be directionally oxidized and polymerized on the surface of HMSNs owing to the electrostatic interaction. The accumulation of PPy on the surface of HMSNs increased with the increase of pyrrole concentration.

Further characterization of MLSs, HMSNs, and PPy/HMSNs was performed by XPS (Figure S2) measurements. In the case of XPS survey spectra, the peaks of oxygen and carbon elements were detected from MLSs, while the peaks of carbon elements disappeared in HMSNs with the appearance of the silicon element. Furthermore, PPy/HMSNs contained not only the peaks of oxygen and silicon elements but also weak peaks of nitrogen and carbon elements, which most probably stemmed from PPy. In addition, the measurement of FTIR spectra (Figure S3) of PPy showed absorption bands at 1544 and 1460 cm^−1^ assigned as pyrrole ring vibrations. C-H in-plane vibrations, C-H stretching vibrations and N-H in-plane vibrations appeared at 1296, 916, and 780 cm^−1^ [12,16,17,18]. In the case of HMSNs, the characteristic peaks of water and silanol OH stretching (3436 cm^−1^), hydrogen bonding of water molecules on silica (1628 cm^−1^), Si-O-Si symmetric stretching (791 cm^−1^), and Si-O asymmetric stretching (1085 cm^−1^) could be observed [1,9,10,11,12,13,14,15,16,17,18,19,20,21]. Moreover, PPy/HMSNs not only had the characteristic peaks of HMSNs but also the characteristic peaks of PPy. Taken together, XPS and FTIR results concluded that PPy was successfully loaded on HMSNs.

Next, the physical properties of the hybrid material were characterized by the N_2_ adsorption–desorption measurement. Figure S4 shows that the HMSNs and PPy/HMSNs maintained IV isotherms and H1 hysteresis loops, indicating that HMSNs had a uniform void structure [22,23]. However, the hysteresis loop of PPy/HMSNs reduced in comparison to only HMSNs. The results indicate that some PPy and PPy molecular segments were filled into the mesopores. In fact, the surface area of HMSNs (554 m^2^·g^−1^) was larger than those of PPy (24.8 m^2^·g^−1^) and PPy/HMSNs (325 m^2^·g^−1^, Table S1), which supported that PPy was successfully wrapped on the surface of HMSNs and the dispersion of PPy was improved. Figure S5 shows the results of the TGA measurements used to investigate the thermal stability of the hybrid material. The weight of HMSNs kept at around 800 °C, while the weight losses of PPy and PPy/HMSNs were estimated to be 32 wt % and 15 wt % at 800 °C, respectively. Thermal decomposition of PPy/HMSNs was observed at 388 °C, which was higher than that of PPy (274 °C). This might be because PPy molecular chains complexed with the inner and outer surfaces of HMSNs through the mesopores. The limited motion of the molecular chains could have improved the thermal stability of PPy/HMSNs. XRD patterns showed that the above samples were amorphous structures with wider diffraction peaks at 20–30° (Figure S6). The negligible difference in the diffraction peaks of PPy/HMSNs and HMSNs indicated that PPy did not change the structure of HMSNs [24].

**Figure 3:**
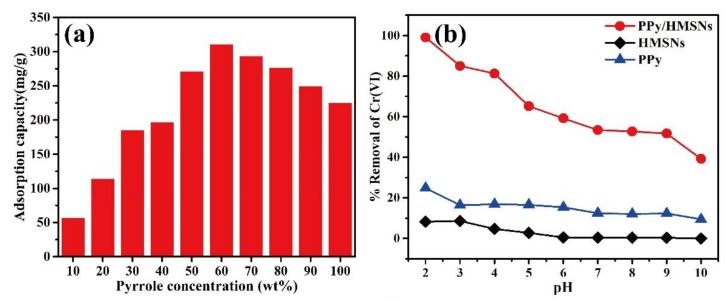
The effect (**a**) of PPy/HMSNs prepared at different pyrrole concentrations on the adsorption capacity of Cr(VI), and the removal rate (**b**) of Cr(VI) by PPy, HMSNs, and PPy/HMSNs at different pH.

The PPy in the composite microspheres has an important role in the adsorption effect. Figure 3a shows the adsorption capability of PPy/HMSNs for Cr(VI) with different pyrrole concentrations. The adsorption capability of PPy/HMSNs increased with increasing PPy concentration and reached the maximum (310.9 mg/g) at 60 wt % of PPy, suggesting that the dispersion of PPy was improved by HMSNs. The adsorption capability decreased with further continuous increases in the ratio of PPy, because an excessive amount of PPy induced the aggregation of PPy and decreased the effective active sites on the surface of PPy/HMSNs. The N_2_ adsorption-desorption curves and pore size distribution curves of PPy/HMSNs showed the specific surface area and pore size were gradually reduced with increasing pyrrole concentrations (Figure S7 and Table S1). In fact, the microscopic images in Figure S8 showed that PPy was uniformly dispersed on the microspheres until the ratio of PPy was less than 60 wt %. In addition, the effect of pH on adsorption is shown in Figure 3b. The removal rate of Cr(VI) by PPy/HMSNs was higher than those of PPy and HMSNs at the range of pH 2−10. The removal rate decreased gradually with the increase in pH because the main chemical species of Cr(VI)-based ionic compounds were changed by pH (pH 2−6: HCrO_4_^−^ and Cr_2_O_7_^2−^, pH 6−8: HCrO_4_^−^ and CrO_4_^2−^, pH > 8: CrO_4_^2−^) [25,26,27]. Furthermore, the zero-potential point of PPy/HMSNs was observed at pH 5.4, as shown in Figure S9. The PPy moieties were protonated above zero-potential (pH < 5.4); thus, the surface of PPy/HMSNs was positively charged and bound to the negatively charged HCrO_4_^−^ through electrostatic interaction, resulting in improvement of the ion exchange performance. With an increase in the pH value (i.e., lower zero potential), the molar ratios of OH^-^ and CrO_4_^2−^ ions are increased, so the competitive binding should decrease the removal efficiency [28,29]. Thus, pH 2.0 was optimal for the removal rate of PPy/HMSNs on Cr(VI). Optimization of the dose of the adsorbent was also carried out. Figure S10 shows the dose-dependent difference in the adsorption behavior for Cr(VI), which showed that the removal rate increased from 52.4% to ~100.0% with an increase in the adsorbent dose. When the removal rate of 25 mL Cr(VI) solution with 400 mg/L initial concentration was ~100.0% at pH 2.0 and 25 °C, the appropriate dose of adsorbent was 50 mg. Overall, these results showed that PPy/HMSNs possessed good adsorption capability for Cr(VI).

**Figure 4:**
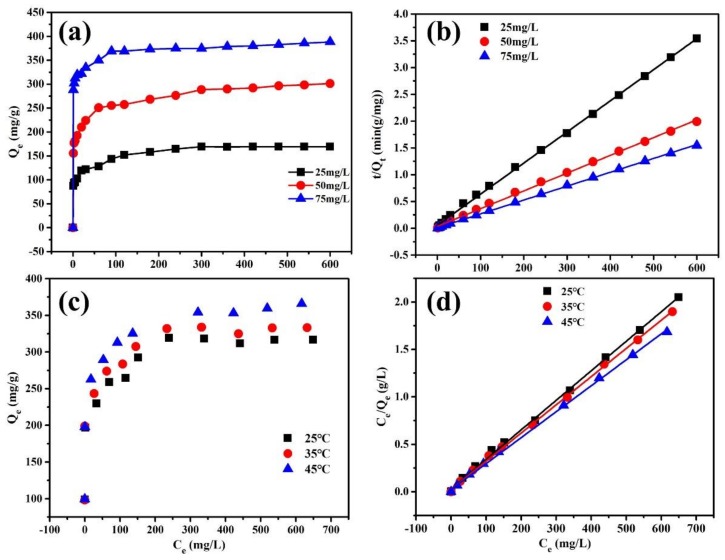
The adsorption kinetics (**a**) and pseudo-second-order fitting curves (**b**) of PPy/HMSNs with different initial concentrations of Cr(VI) solution, adsorption isotherms (**c**) and Langmuir model fitting curves (**d**) of PPy/HMSNs with different temperature.

The adsorption kinetics and isotherms were further studied to analyze the adsorption process of Cr(VI) by PPy/HMSNs, and the corresponding modeling results and parameters are summarized in Figure 4, Figures S11 and S12 and Tables S2 and S3. In the different initial Cr(VI) concentrations, the adsorption capability was increased with the increase in the adsorption time. The adsorption capabilities were estimated to be 169, 294, and 382 mg/g at different initial Cr(VI) concentrations (25, 50, and 75 mg/L). Quasi-first-order and quasi-second-order models were used to fit the adsorption process, which showed that the *R*^2^ of the quasi-second-order kinetic model were closer to 0.999 compared with that of the quasi-first-order kinetic model (0.829, 0.945, 0.820) at different initial Cr(VI) concentrations. Moreover, the adsorption process was also fitted by the intra-particle diffusion model (Figure S11b) to analyze the properties of rate control in the adsorption process. The calculated results showed that the adsorption process had three stages. The larger slope of the first stage revealed that the high adsorption rate was due to boundary film diffusion, which was the diffusion process of Cr(VI) to the surface of the adsorbents through the aqueous solution. The gentle slope of the second stage indicated that the adsorption rate was low, which was due to the internal diffusion or pore diffusion of particles in the PPy/HMSNs. The third stage meant the equilibrium stage [30,31].

Figure 4c,d shows the adsorption isotherms of Cr(VI) by PPy/HMSNs at different temperatures and the fitting curves using the Langmuir model. The fitting curves of the Freundlich model and parameters are shown in Figure S12 and Table S3. The fitting results showed that the adsorption process was better expressed by the Langmuir model (*R*^2^ = 0.999). Moreover, *R*_L_ was between 0 and 1, indicating that the adsorption of Cr(VI) by PPy/HMSNs was a favorable process [32,33,34]. For the 1/*n* of Freundlich, values less than 0.5 demonstrated that the adsorbent could easily adsorb Cr(VI). As the temperature increased, the smaller and smaller 1/*n* values showed that the high temperature was more favorable for adsorption [35,36,37]. The maximum adsorption capacity (*Q*_m_) was 322 mg/g at 25 °C in the Langmuir model, which was larger than that of most adsorbents (Table S4).

**Figure 5:**
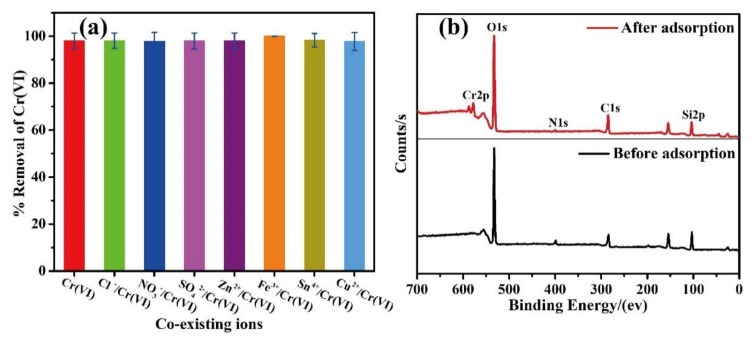
(**a**) The effect of coexisting ions on Cr(VI) adsorption by PPy/HMSNs and (**b**) XPS spectra of PPy/HMSNs before and after adsorption.

Further, Figure S13 shows the adsorption capacities at different temperatures and plots of ln*Q*_e_/*C*_e_ against 1/*T* for the adsorption of Cr(VI) onto the PPy/HMSNs adsorbent. The adsorption thermodynamic parameters are shown in Table S5. The Gibbs free energy (∆*G*^0^) was negative at different temperatures, suggesting that the adsorption process was spontaneous and feasible [7,38,39,40]. In the actual environment, wastewater does not contain only Cr(VI). Thus, this study further discusses the effects of coexisting ions on the adsorption of Cr(VI) by PPy/HMSNs (Figure 5a). The removal rate of Cr(VI) by PPy/HMSNs was ~100% in the binary systems of Cl^−^/Cr(VI), NO_3_^−^/Cr(VI), SO_4_^2−^/Cr(VI), Zn^2+^/Cr(VI), Fe^3+^/Cr(VI), Sn^4+^/Cr(VI), and Cu^2+^/Cr(VI), indicating no competition between the coexisting ions and Cr(VI) on the surface of the adsorbents. Cyclic adsorption properties were also discussed (Figure S14). Considering the cyclic adsorption performance of the adsorbents, the removal rate of Cr(VI) was still more than 97% after five times of cyclic adsorption–desorption. The results showed that PPy/HMSNs was an excellent absorbent for the removal of Cr(VI) in water.

The EDS data (Figure S15), elemental mapping micrographs (Figure S16), and XPS curves (Figure 5b) of PPy/HMSNs before and after adsorption were determined to further study the adsorption phenomena of PPy/HMSNs for the adsorption of Cr(VI). The EDS data show that the characteristic peak of Cr(VI) appeared and the characteristic peak of Cl^−^ disappeared after the adsorption. In the elemental mapping micrographs, Cr(VI) was uniformly adsorbed on the surface of PPy/HMSNs, which was due to the ion exchange between Cl^−^ and Cr(VI). Moreover, the appearance of Cr(VI) characteristic peaks and weakened Cl^−^ characteristic peaks in the XPS curves after adsorption further suggest that Cr(VI) was adsorbed by PPy/HMSNs. The Cr 2p peak in XPS data (Figure S17) shows that the characteristic peaks of Cr(III) and Cr(VI) were clearly observed after the adsorption, confirming that PPy/HMSNs possessed strong reduction ability and reduced Cr(VI) to Cr(III) [41].

## 4. Conclusions

In summary, the novel adsorbent of PPy/HMSNs composite was synthesized by in-situ polymerization. In the composite, HMSNs could effectively solve the agglomeration problem of PPy. Good dispersion of PPy provided more active sites for the adsorption of Cr(VI). The adsorption process could be well fitted to the quasi-second-order kinetic model and the Langmuir isotherm model. The maximum adsorption capacity of Cr(VI) was 322 mg/g. In addition, the removal rate of Cr(VI) was almost unaffected by other ions. After five cycles of adsorption–desorption, the removal rate of Cr(VI) was still more than 97%. Moreover, the adsorption mechanism further showed that PPy/HMSNs had strong reduction ability and could reduce high-toxicity Cr(VI) to low-toxicity Cr(III). Therefore, the PPy/HMSNs composite is a new adsorbent with promising potential for the removal of Cr(VI) from wastewater.

## Supplementary Materials

The following are available online at https://www.mdpi.com/2079-4991/10/4/686/s1, The adsorption experiment details. Table S1: specific surface area and aperture parameters of PPy, HMSNs, and PPy/HMSNs with different pyrrole concentrations. Tables S2 and S3 show the adsorption kinetic and isothermal parameters of the adsorption of Cr(VI) onto the PPy/HMSNs. The performance comparison of PPy/HMSNs and other adsorbents for removing Cr(VI) at 25 °C is shown in Table S4. Table S5 shows adsorption thermodynamic parameters of PPy/HMSNs. Figure S1. shows the standard curve line of Cr(VI) solutions. Figure S2 shows the XPS survey curves of MLSs, HMSNs, and PPy/HMSNs. The FTIR spectra, N_2_ adsorption-desorption curves, and pore size distribution curves, thermogravimetric curves, and XRD patterns of PPy, HMSNs, and PPy/HMSNs are shown in Figures S3-S6, respectively. Figures S7 and S8 show the N_2_ adsorption-desorption curves, pore size distribution curves, and SEM images of PPy/HMSNs with different pyrrole concentrations. Figure S9 shows Zeta potential of PPy, HMSNs, and PPy/HMSNs at different pHs. Figure S10 shows effects of different adsorbent doses on the adsorption of Cr(VI). Figures S11 and S12 show the adsorption kinetics and isotherm fitting curves of Cr(VI). Figure S13 shows adsorption capacities of PPy/HMSNs for Cr(VI) at different temperature, and plots of lnQ_e_/C_e_ against 1/T. Removal efficiency of PPy/HMSNs in five cycles of sorption/desorption for Cr(VI) is shown in Figure S14. Figure S15 shows EDS spectrum of PPy/HMSNs before and after adsorption. Figures S16 and S17 show the elemental mapping micrographs and Cr2p XPS profiles of PPy/HMSNs after adsorption.
