# Supplementary material for: Removal of Cr(VI) from Aqueous Solution by Polypyrrole/Hollow Mesoporous Silica Particles
Source: Nanomaterials (Basel). 2020 Apr 5;10(4):686. doi: 10.3390/nano10040686 (PMC7221785; doi:10.3390/nano10040686)
Supplement: Supplementary file 1 [file nanomaterials-10-00686-s001.pdf]

Supplementary Materials

Removal of Cr(VI) from Aqueous Solution by Polypyrrole/Hollow Mesoporous Silica Particles

Linlin Du [1], Peng Gao [1], Yuanli Liu [1,*] Tsuyoshi Minami [2] and Chuanbai Yu [1,*]

[1] Guangxi Key Laboratory of Optical and Electronic Materials and Devices, College of Materials Science and Engineering, Guilin University of Technology, Guilin 541004, China; linlindu0916@163.com (L.D.); pgaoglut@163.com (P.G.)

[2] Institute of Industrial Science, the University of Tokyo, 4-6-1 Komaba, Meguro-ku 153-8505, Tokyo, Japan; tminami@iis.u-tokyo.ac.jp

* Correspondence: lyuanli@glut.edu.cn (Y.L.); ycb2008@glut.edu.cn (C.Y.)

Adsorption experiments

For the determination of Cr(VI) concentration in solution, Cr(VI) aqueous solutions with certain concentration (0.05, 0.1, 0.2, 0.4, 0.6, 0.8 and 1.0 mg/L) were first prepared according to the *national standard of the people's Republic of China* (GB7467-87). Then, the absorption spectrums of the solutions were measured by the ultraviolet-visible spectrophotometer. And Cr(VI) solutions had an obvious absorption peak at 540 nm. Therefore, the standard curve of Cr(VI) solutions was determined according to the certain concentration and absorbance of solutions at 540 nm (Figure S1). Then, the Cr(VI) concentration of unknown solutions was obtained by measuring the absorbance at 540 nm using PerkinElmer instrument and standard curve.

Adsorption properties of PPy/HMSNs prepared with different Py concentrations

PPy/HMSNs (25 mg, Py concentrations: 10~100 wt%) was added to the Cr(VI) solution (50 mL) with an initial concentration of 200 mg/L at pH 2.0 for 24 h at 25 °C. Then, the mixture was filtered to measure the content of Cr(VI). And the adsorption capacity of Cr(VI) was calculated using Eq. 1 to determine the optimum Py concentration.

$$Q_e=\frac{(C_0-C_e)}{m}V \quad (1)$$

where Q_e is the mass of Cr(VI) absorbed by adsorbents per unit mass (mg·g^{-1}), C_0 is the initial Cr(VI) concentration (mg·L^{-1}), C_e is the Cr(VI) concentration after adsorption (mg·L^{-1}), V is the volume of the solution (mL) and m is the weight of the adsorbent (mg).

Adsorption properties of PPy/HMSNs at different pH

The adsorbents (25 mg) was added to the Cr(VI) solution (25 mL) with an initial concentration of 100 mg/L at different pH (2.0~10) for 24 h at 25 °C. Then, the mixture was filtered to measure the content of Cr(VI). And the removal rate of Cr(VI) was calculated using Eq. 2 to determine the optimum pH.

$$\%\ \text{Removal}=\frac{(C_0-C_e)}{C_0}V\times 100 \quad (2)$$

Adsorption properties of PPy/HMSNs with different dose

The PPy/HMSNs (10~100 mg) was added to the Cr(VI) solution (25 mL) with an initial concentration of 400 mg/L at pH 2.0 for 24 h at 25 °C. Then, the mixture was filtered to measure the content of Cr(VI). And the removal rate and adsorption capacity of Cr(VI) was calculated using Eq. 1and Eq. 2 to determine the optimum dose.

Influence of co-existing ions for the Cr(VI) adsorption

The PPy/HMSNs (20 mg) was added to 20 mL of the binary solution system (the concentration of Cr(VI), Cl^-, NO_3^-, SO_4^{2-}, Zn^{2+}, Fe^{3+}, Sn^{4+} and Cu^{2+} were all 100 mg/L) at pH 2.0 for 24 h at 25 °C. Then, the mixture was filtered to measure the content of Cr(VI). And the removal rate of Cr(VI) was calculated using Eq. 2.

Cyclic adsorption

The PPy/HMSNs (50 mg) was added to the Cr(VI) solution (20 mL) with an initial concentration of 100 mg/L at pH 2.0 and 25 °C. After adsorption, the mixture was filtered. Then, the adsorbent was desorbed using NaOH solution (0.1 mol/L) for 3 h and washed using H_2O. Finally, the adsorbent was dried at 60 °C for 6 h, and the adsorption-desorption experiments were repeated 5 times.

Adsorption kinetics

The adsorbents (30 mg) was added to the Cr(VI) solution (200 mL) with different initial concentration (25, 50 and 70 mg/L) at pH 2.0 and 25 °C. Then, some mixture solutions were collected and filtered to measure the concentration of Cr(VI) at time t. And the adsorption capacity (Q_t) of Cr(VI) at time t was calculated using Eq. 3. Finally, the data were fitted by quasi-first-order kinetic (Eq. 4), quasi-second-order kinetic (Eq. 5) and particle diffusion (Eq. 6) models.

$$Q_t=\frac{(C_0\text{-}C_t)}{m}V \tag{3}$$

$$\ln(Q_e\text{-}Q_t)=\ln Q_e-\frac{K_1 t}{2.303} \tag{4}$$

$$\frac{t}{Q_t}=\frac{1}{K_2Q_e^2}+\frac{t}{Q_e} \tag{5}$$

$$Q_t=K_{ip}t^{0.5}+C \tag{6}$$

where Q_t is the mass of Cr(VI) absorbed by adsorbents per unit mass ($mg\cdot g^{-1}$) at time t, C_t is the Cr(VI) concentration after adsorption ($mg\cdot L^{-1}$) at time t, K_1 is the pseudo-first-order kinetic adsorption rate constant (min^{-1}), K_2 is the pseudo-second-order kinetic adsorption rate constant ($g\cdot mg^{-1}\cdot min^{-1}$), K_{ip} is the particle diffusion model adsorption rate constant ($mg\cdot g^{-1}\cdot min^{0.5}$), and C is the intercept related to the boundary layer thickness.

Adsorption isotherms

The adsorbents (25 mg) was added to the Cr(VI) solution (50 mL) with different initial concentration (50~800 mg/L) at pH 2.0 and different temperature (25, 35 and 45 ℃). Then, some mixture solutions were collected and filtered to measure the concentration of Cr(VI). And the adsorption data were fitted by Langmuir (Eq. 7) and Freundlich (Eq. 9) models.

$$\frac{C_e}{Q_e}=\frac{C_e}{Q_m}+\frac{1}{bQ_m} \tag{7}$$

$$R_L=\frac{1}{1+bC_0} \tag{8}$$

$$\ln Q_e=\ln K+\frac{1}{n}\ln C_e \tag{9}$$

where Q_m is the maximum adsorption capacity ($mg\cdot g^{-1}$), b is the adsorption free energy constant ($L\cdot mg^{-1}$), R_L is a nondimensional factor, the Freundlich constant (K) indicates the relative adsorption capacity of the adsorbents ($mg\cdot g^{-1}$), 1/n is the adsorption strength.

Adsorption thermodynamics

The Gibbs free energy change (ΔG^0), enthalpy change (ΔH^0) and entropy change (ΔS^0) are the major parameters in the adsorption process. The parameters are calculated by the Eqs. 10-12.

$$K_c=(C_0-C_e)\frac{V}{mC_e} \tag{10}$$

$$\ln K_c=\frac{\Delta S^0}{R}-\frac{\Delta H^0}{RT} \tag{11}$$

$$\Delta G^0=-RT\ln K_c \tag{12}$$

where R is the universal gas constant (8.314 $J\cdot mol^{-1}\cdot K^{-1}$) and T is the thermodynamic temperature (K).

Table S1. Specific surface area and aperture parameters of PPy, HMSNs and PPy/HMSNs with different pyrrole concentrations.

Sample	BET Surface area(m^2/g)	Pore Volume(cm^3/g)	Average pore(nm)
HMSNs	553.97	1.01	7.31
HMSNs-PPy (20 wt%)	460.54	0.79	7.87
HMSNs-PPy (40 wt%)	354.88	0.69	7.81
HMSNs-PPy (60 wt%)	325.00	0.62	7.64
HMSNs-PPy (80 wt%)	278.81	0.50	7.30
HMSNs-PPy (100 wt%)	259.93	0.47	7.18
PPy	24.82	/	/

Table S2. Adsorption kinetic parameters of the adsorption of Cr(VI) onto the PPy/HMSNs adsorbent at pH 2.0 and 25 °C. The initial Cr(VI) concentration was 25, 50 and 75 mg/L.

	Q_{e-exp}	Pseudo-second-order			Pseudo-first-order			Intraparticle diffusion		
		$t/Q_t=1/K_2Q_e^2+t/Q_e$			$\ln(Q_e-Q_t)=\ln Q_e-K_1t/2.303$			$Q_t=K_{ip}t^{0.5}+C$		
		Q_{e-cal}	K_2	R^2	Q_{e-cal}	K_1	R^2	K_{ip}	C	R^2
25 mg/L	169.17±0.14	172.12	5.98×10^{-4}	0.999	79.04	0.0147	0.829	4.49	82.04	0.695
50 mg/L	294.43±5.08	301.20	3.14×10^{-4}	0.999	119.10	0.00676	0.945	7.32	152.15	0.652
75 mg/L	381.64±4.93	386.10	6.29×10^{-4}	0.999	76.71	0.00614	0.820	6.60	259.64	0.349

Table S3. Isothermal parameters of adsorption for Cr(VI) by PPy/HMSNs at 298 K, 308 K and 318 K.

Isotherm model	Parameters	298 K	308 K	318 K
Langmuir	Q_m	321.54	336.70	366.30
	B	0.102	0.119	0.144
	R_L	0.164	0.144	0.145
	R^2	0.999	0.999	0.999
Freundlich	K	168.928	191.080	208.350
	1/n	0.104	0.091	0.088
	R^2	0.968	0.834	0.822

Table S4. Performance comparison of PPy/HMSNs and other adsorbents for removing Cr(VI) at 25 °C.

Adsorbents	Q_m (mg/g)	Equilibrium time (min)	Optimum pH	References
Polypyrrole-polyaniline nanofibers	227	30-180	2.0	Bhaumik[1]
Polypyrrole/wood sawdust	3.4	10-15	5.0	Ansari[2]
Polypyrrole/Fe_3O_4 magnetic nanocomposite	169.4	30-180	2.0	Bhaumik[3]
Polypyrrole/graphene oxide composite	497.1	180-1440	3.0	Li[4]
Polyacrylonitrile/polypyrrole core/shell nanofiber mats	61.8	30-90	2.0	Wang[5]
Fe_3O_4@glycine-polypyrrole magnetic nanocomposites	238	30-180	2.0	Ballav[6]
Polypyrrole -organically modified montmorillonite clay	119.3	180	2.0	Setshedi[7]
Surfactant-modified zeolite	5.19	/	6.0	Leyva-Ramos[8]
PPy/HMSNs	321.54	100	2.0	[Present study]

Table S5. Adsorption thermodynamic parameters of PPy/HMSNs.

Temperature (°C)	ΔG^0 (J/mol)	ΔH^0 (J/mol)	ΔS^0 (J/mol/K)
25	-10.218		
35	-13.038	73.818	0.282
45	-15.858		

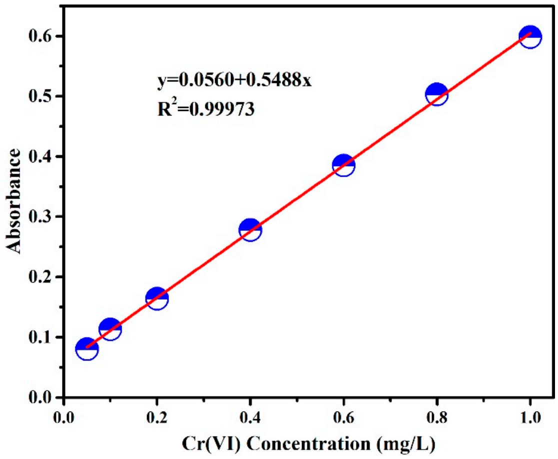

Figure S1. The standard curve line of Cr(VI) solutions.

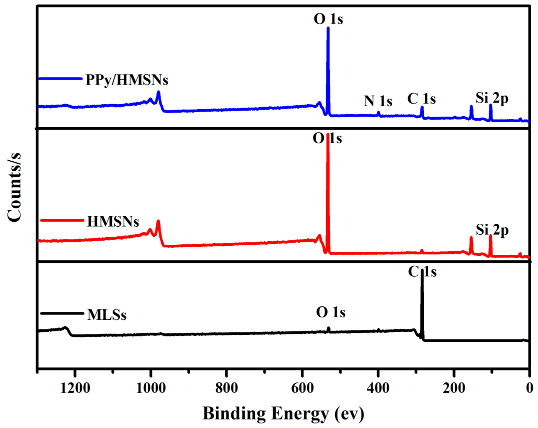

Figure S2. The XPS survey curves of MLSs, HMSNs and PPy/HMSNs.

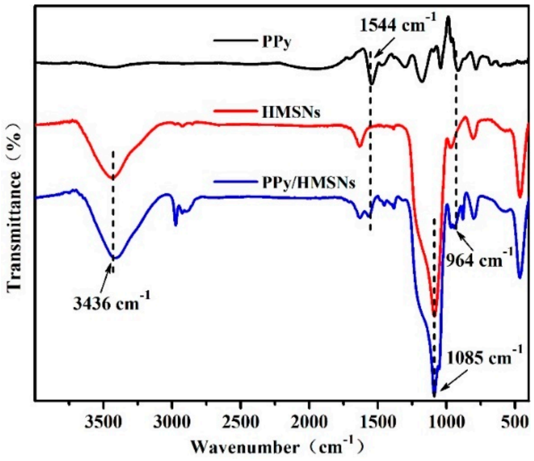

Figure S3. The FT-IR spectra of PPy, HMSNs and PPy/HMSNs.

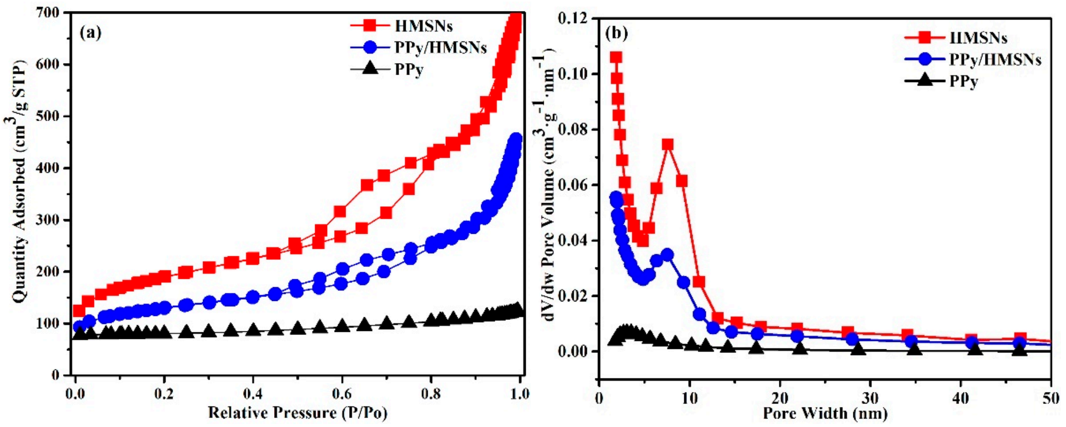

Figure S4. The N_2 adsorption-desorption curves (a) and pore size distribution curves (b) of PPy, HMSNs and PPy/HMSNs.

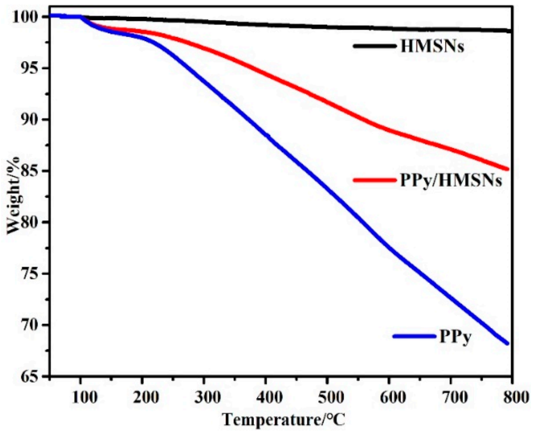

Figure S5. The thermogravimetric curves of HMSNs, PPy/HMSNs and PPy.

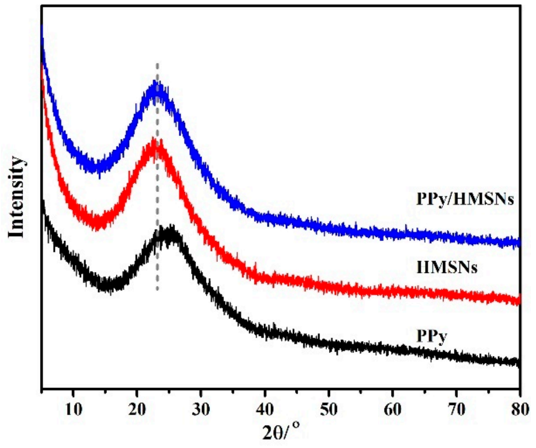

Figure S6. The XRD patterns of HMSNs, PPy/HMSNs and PPy.

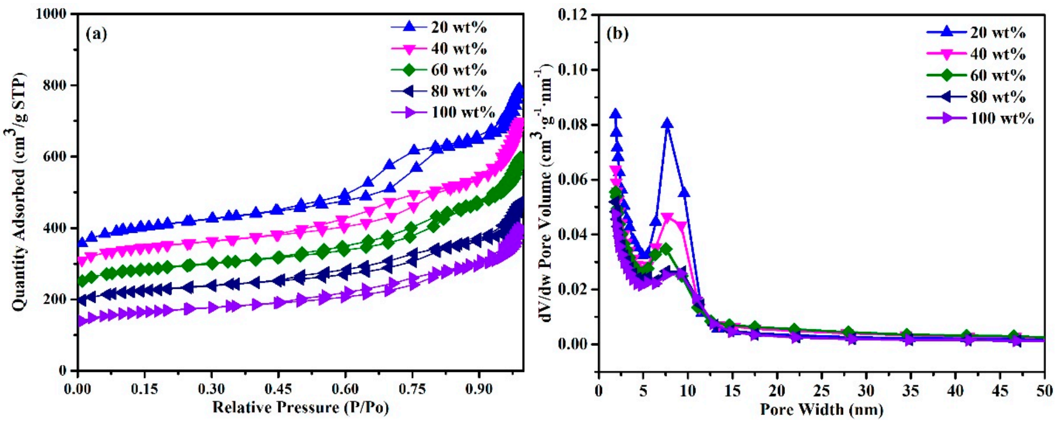

Figure S7. The N_2 adsorption-desorption curves (a) and pore size distribution curves (b) of PPy/HMSNs with different pyrrole concentrations (20 wt%, 40 wt%, 60 wt%, 80 wt% and 100 wt%).

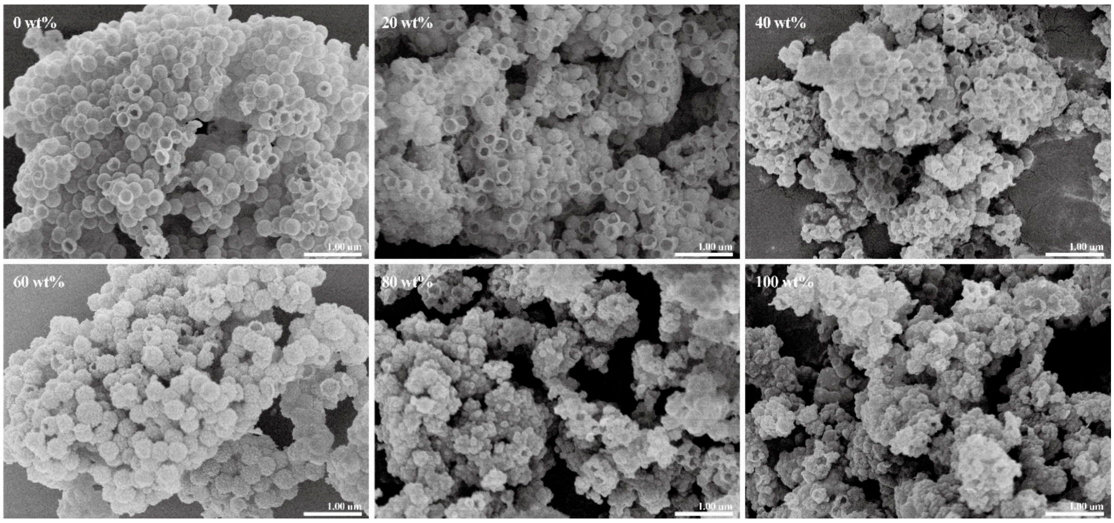

Figure S8. The SEM images of PPy/HMSNs with different pyrrole concentrations (0 wt%, 20 wt%, 40 wt%, 60 wt%, 80 wt% and 100 wt%).

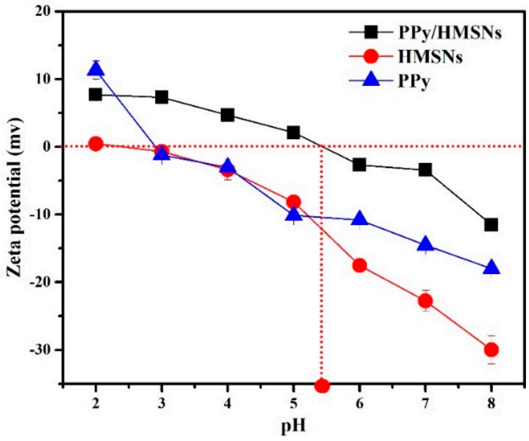

Figure S9. Zeta potential of PPy, HMSNs and PPy/HMSNs at different pH.

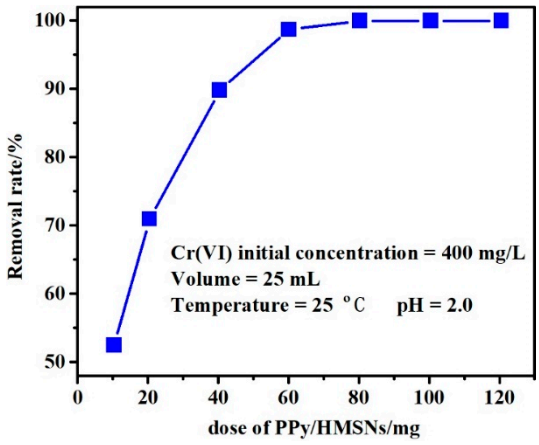

Figure S10. Effect of different adsorbent dose on the adsorption of Cr(VI).

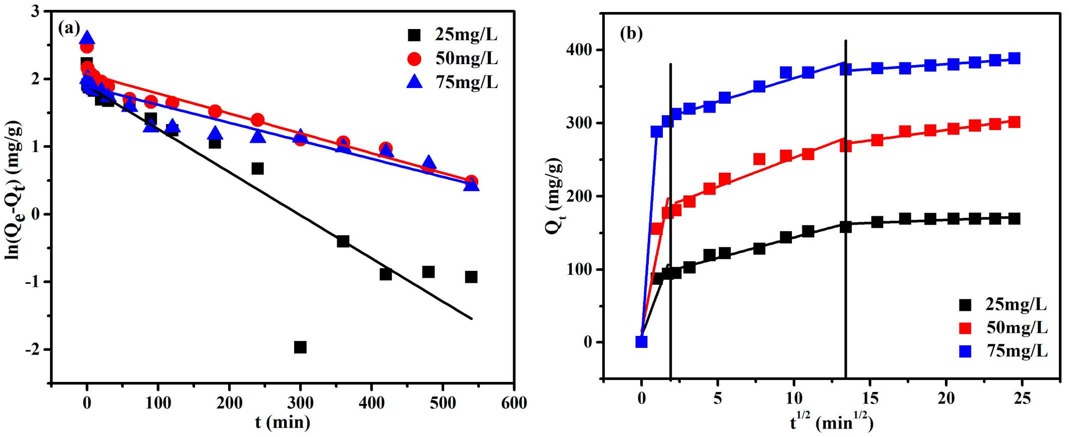

Figure S11. Fitting the adsorption kinetics of Cr(VI) onto PPy/HMSNs using (a) pseudo-first-order, and (b) intra-particle diffusion models.

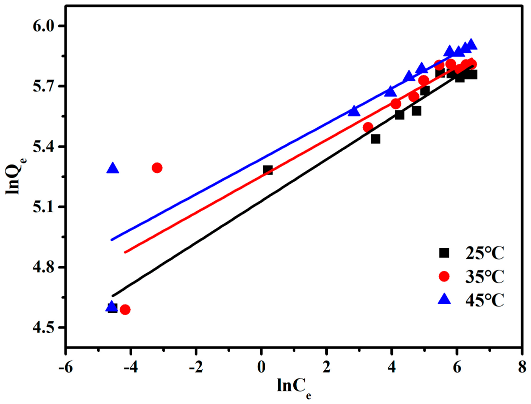

Figure S12. The adsorption isotherms of Cr(VI) onto PPy/HMSNs fitting curves by Freundlich model.

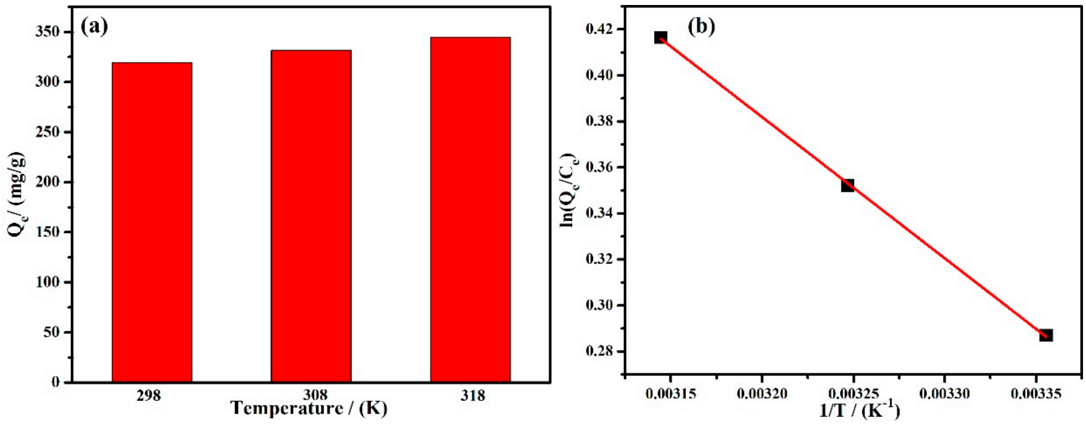

Figure S13. Adsorption capacities of PPy/HMSNs for Cr(VI) at different temperature(a), and plots of $\ln Q_e/C_e$ against 1/T for the adsorption of Cr(VI) onto the PPy/HMSNs adsorbent.

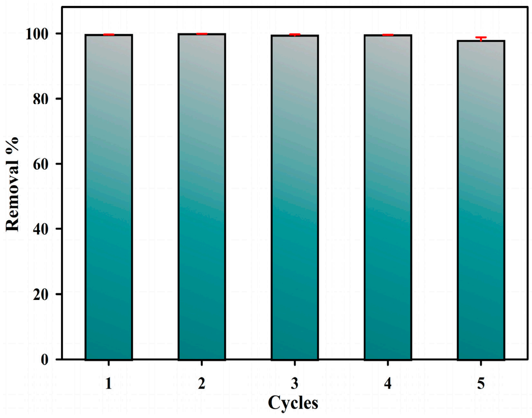

Figure S14. Removal efficiency of PPy/HMSNs in five cycles of sorption/desorption for Cr(VI).

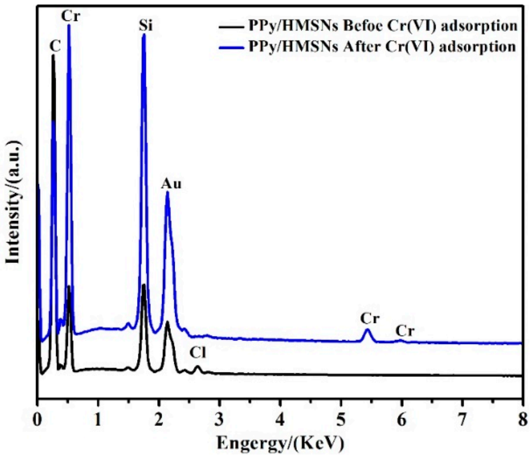

Figure S15. The EDS spectrum of PPy/HMSNs before and after adsorption.

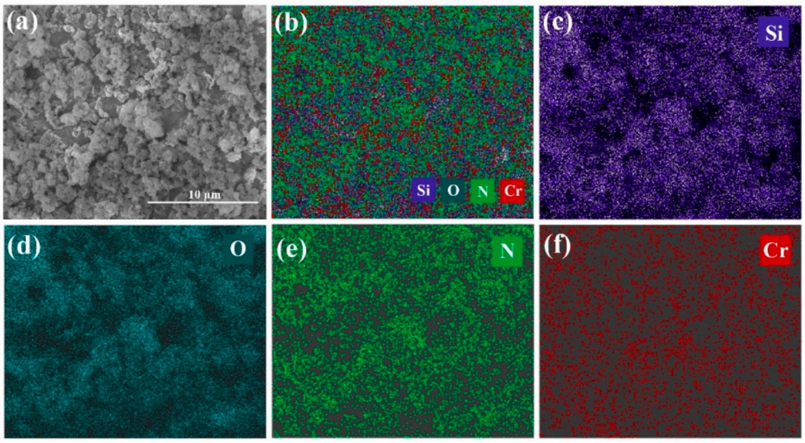

Figure S16. The elemental mapping micrographs of overall (a, b), silicon (c), oxygen (d), nitrogen (e) and chromium (f) after PPy/HMSNs adsorption for Cr(VI).

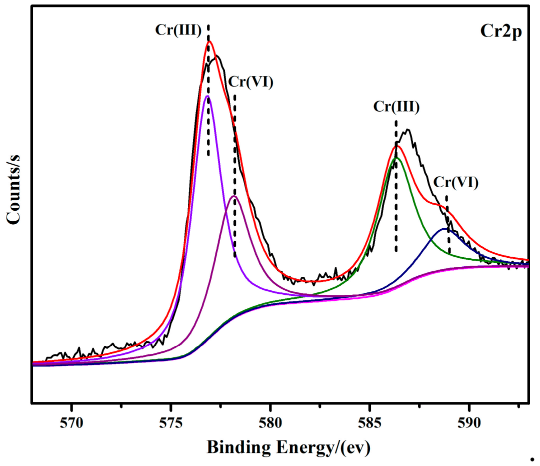

Figure S17. The Cr2p XPS profile of PPy/HMSNs after adsorption.

References

1. Bhaumik, M.; Maity, A.; Srinivasu, V.V.; Onyango, M.S. Removal of hexavalent chromium from aqueous solution using polypyrrole-polyaniline nanofibers. *Chem. Eng. J.* **2012**, *181-182*, 323-333.
2. Ansari R.; Fahim, N.K. Application of polypyrrole coated on wood sawdust for removal of Cr (VI) ion from aqueous solutions. *React. Funct. Polym.* **2007**, *67*, 367–374.
3. Bhaumik, M.; Maity, A.; Srinivasu, V.V.; Onyango, M.S. Enhanced removal of Cr(VI) from aqueous solution using polypyrrole/Fe_3O_4 magnetic nanocomposite. *J. Hazard. Mater.* **2011**, *190*, 381–390.
4. Li, S.; Lu, X.; Xue, Y.; Lei, J.; Zheng, T.; Wang, C. Fabrication of polypyrrole/graphene oxide composite nanosheets and their applications for Cr(VI) removal in aqueous solution. *PLoS ONE* **2012**, *7*, e43328.
5. Wang, J.; Pan, K.; He, Q.; Cao, B. Polyacrylonitrile/polypyrrole core/shell nanofiber mat for the removal of hexavalent chromium from aqueous solution. *J. Hazard. Mater.* **2013**, *244-245*, 121-129.
6. Ballav, N.; Choi, H.J.; Mishra, S.B.; Maity, A. Synthesis, characterization of Fe3O4@ glycine doped polypyrrole magnetic nanocomposites and their potential performance to remove toxic Cr(VI). *J. Ind. Eng. Chem.* **2014**, *20*, 4085–4093.
7. Setshedi, K.Z.; Bhaumik, M.; Songwane, S.; Onyango, M.S.; Maity, A. Exfoliated polypyrrole-organically modified montmorillonite clay nanocomposite as a potential adsorbent for Cr (VI) removal. *Chem. Eng. J.* **2013**, *222*, 186–197.
8. Leyva-Ramos, R.; Jacobo-Azuara, A.; Diaz-Flores, P.E.; Guerrero-Coronado, R.M.; Mendoza-Barron, J.; Berber-Mendoza, M.S. Adsorption of chromium(VI) from an aqueous solution on a surfactant-modified zeolite. *Colloids Surf., A* **2008**, *330*, 35–41.
